# Assessment of Evidence Regarding Minimally Invasive Surgery vs. Conservative Treatment on Intracerebral Hemorrhage: A Trial Sequential Analysis of Randomized Controlled Trials

**DOI:** 10.3389/fneur.2020.00426

**Published:** 2020-06-04

**Authors:** Xiang Zhou, Li Xie, Yuksel Altinel, Nidan Qiao

**Affiliations:** ^1^Department of Neurosurgery, Shanghai Pituitary Tumor Center, Shanghai Neurosurgical Research Institute, Huashan Hospital, Shanghai Medical College, Fudan University, Shanghai, China; ^2^Nursing Department, Huashan Hospital, Shanghai Medical College, Fudan University, Shanghai, China; ^3^Medical Science in Clinical Investigation, Harvard Medical School, Boston, MA, United States; ^4^Department of Neurosurgery, Huashan Hospital North Campus, Shanghai Medical College, Fudan University, Shanghai, China; ^5^Neuroendocrine Unit, Massachusetts General Hospital, Harvard Medical School, Boston, MA, United States

**Keywords:** endoscope, stereotactic evacuation, thrombolysis, stroke, meta-analysis, mortality

## Abstract

**Introduction:** The recent publication of a trial failed to prove the efficacy of minimally invasive surgery (MIS) in patients with intracerebral hemorrhage. The aim of this study was to answer the question: Do we need more trials to compare MIS vs. conservative treatment in these patients?

**Methods:** Databases were searched for relevant randomized trials on MIS (endoscopic surgery or stereotactic evacuation) vs. conservative treatment. The primary outcome was significant neurological debilitation or death at the follow-up, and the secondary outcome was death. Both conventional meta-analysis and trial sequential analysis (TSA) were performed.

**Results:** Twelve trials with 2,049 patients were included. In the conventional meta-analysis, the risk ratios of MIS vs. conservative treatment were 0.82 [95% confidence interval (CI), 0.72–0.94] and 0.74 (95% CI, 0.62–0.88) for the primary and secondary outcomes, respectively. In TSA, the cumulative *z* curve crossed the superiority boundary, which confirmed an 18.8% relative risk reduction of MIS vs. conservative treatment for the primary outcome. It was also highly likely that MIS would reduce mortality by 24.3%. Several sensitivity analyses suggested the robustness of our results, including different prior settings, including only trials with blind outcome assessment, and the assumption of future trials to be futile.

**Conclusions:** Minimally invasive surgery seems to be more effective than conservative treatment in patients with intracerebral hemorrhage in reducing both morbidity and mortality. Repeating a clinical trial with similar devices, design, and outcomes is unlikely to change the current evidence.

## Introduction

Stroke contributes 5% to all disability-adjusted life-years loss (1) and 10% to all deaths worldwide (2). Hemorrhagic stroke accounts for more disability-adjusted life-years loss than ischemic stroke (1). In theory, surgical evacuation of hemorrhage may improve the patient's outcome, but several randomized trials (3, 4) failed to prove its effectiveness. Morbidity and mortality remained high even in patients treated. With the development of endoscopic and stereotactic evacuation technique, more and more patients were treated with these minimally invasive surgeries (MISs).

The arguments of the benefit by MIS were controversial among several randomized trials; nearly half of the published trials were futile. Several previous systematic reviews and meta-analyses of MIS include retrospective or prospective nonrandomized studies with potential confounding and bias (5–7). The results of the meta-analyses on randomized trials also varied from “nonsuperiority” (8) to “superiority” (9, 10) with different outcome measurements and different control group selections. The recent publication of a phase 3 trial failed to prove the efficacy of MIS, which made the question more suspicious (11).

The question still lies in which treatment is better for patients with intracerebral hemorrhage: MIS or conservative treatment. Do we need more trials to compare MIS vs. conservative treatment in these patients? Trial sequential analysis (TSA) borrows the idea from interim monitoring from a single randomized trial by treating every trial in the meta-analysis as an interim sample (12). Similar to interim monitoring, TSA has rules for early stopping if the result meets superiority boundary or futility boundary. In this review, we aimed to apply TSA on data from randomized trials comparing MIS vs. conservative treatment to answer the questions as mentioned above.

## Methods

### Search Strategy and Selection Criteria

This study is reported according to PRISMA (Preferred Reporting Items for Systematic Reviews and Meta-Analyses) guidelines (Supplement Table 1). We searched randomized controlled trials (RCTs) published up to March 1, 2019, using the combination of stroke (“intracranial hemorrhage,” “intracerebral hemorrhage,” “cerebral hemorrhage,” “brain hemorrhage,” “stroke”) and surgical modality (“endoscope,” “evacuation,” “minimally”) in PubMed, Web of Science, and China Knowledge Resource Integrated Database (detailed strategy in Supplement Table 2). We screened for additional eligible trials in reference lists of retrieved studies and relevant review articles. The exclusion criteria were as follows: studies with brain hemorrhage due to traumatic brain injury, tumor, coagulopathy, or vascular disease; studies with both craniotomy and MIS, but the decision of craniotomy or MIS was made at the discretion of surgeons; nonrandomized studies; and trials in which outcome information was not available.

### Type of Interventions

Minimally invasive surgery comprised endoscopic surgery or stereotactic evacuation with or without thrombolysis. Conservative treatment was the best conventional medical treatment.

### Outcomes

The primary outcome was the proportion of patients with significant neurological debilitation or death at the postrandomization follow-up. Significant neurological debilitation or death was defined as modified Rankin Score of more than 3 or Glasgow Outcome Scale of <4. As we expected the literature to be heterogeneous in terms of follow-up duration, we adopted the primary outcome time point reported in the original trial. The secondary outcome was the proportion of patients who died at the postrandomization follow-up. Regarding crossover in the included trials, we used the intention-to-treat effect. We also imputed the loss to follow-up data as the worst outcome.

### Data Extraction

X.Z. and L.X. independently screened the literature, selected studies, extracted the relevant information, and assessed the risk of bias with the Cochrane risk of bias tool (13). Risk of bias for each item was classified as either low risk, unclear, or high risk. Any controversies were resolved by consensus and arbitration by the entire review team including a senior consultant physician (N.Q.).

### Data Synthesis and Statistical Methods

Outcomes were recorded as the proportion in each arm. A conventional meta-analysis was used to pool risk ratios comparing MIS with conservative treatment. We initially used random-effects models to aggregate data and the *I*^2^ tests to examine heterogeneity [more than 50% indicates notable heterogeneity (14)]. When no significant heterogeneity was observed, we changed our models into fixed-effects models. We performed subgroup analysis by different mean ages (<60 or >60 years old), follow-up period (≤ 1 year), study quality (blind or unblind outcome assessment), publication year (before 2010 or after 2010), study location (Eastern Asia or Western), and surgical modality (endoscopic surgery or stereotactic evacuation).

We conducted a TSA assuming 5% as an acceptable risk of type I error (α). We set several prior to the TSA: (1) effect size: we selected an 18.8% relative risk reduction as *a priori*, which was estimated from the conventional meta-analysis; (2) statistical power: we chose 80%; (3) event proportion in the control arm: we used 67.4% as an estimate from the pooled primary outcome from all the control groups; (4) amount of heterogeneity: 81.9% as the observed diversity across the included trials. For the secondary outcome, we used the same procedures with the prior obtained from the currently available evidence. The TSA combines the information size (cumulated sample size) with trial sequential monitoring boundaries. Whether the conclusion is sufficient was determined based on the following criteria: the evidence is adequate when the cumulative *z* curve crosses the monitoring boundary, and the evidence is insufficient if the *z* curve does not intersect any of the boundaries, and the required information size has not been reached (15).

We performed several sensitivity analyses. First, we used more conservative analyses prior, such as a reduced risk reduction (15 and 10%), an increased power (90%), or a decreased event proportion in the control arm (58.0%) according to the most recent trial (11). Second, we repeated the analysis only in trials with high quality (blind outcome assessment). Third, we further assumed the result of the ongoing RCT (NCT02880878) to be futile to discern the impact on the analysis.

Statistical analyses were performed with RStudio version 1.0.143 (Boston, MA) for the conventional meta-analysis and Trial Sequential Analysis software 0.9 (Copenhagen Trials Unit, Copenhagen, Denmark) for the TSA.

## Results

Twelve trials (11, 16–26) were included with nine trials in English and three in Chinese. Flowchart of inclusion and exclusion is provided in Supplement Figure 1, and baseline characteristics of the included studies are listed in Supplement Table 3. There were 2,049 patients included in this study. Nine trials investigated the effect of stereotactic evacuation, and three trials examined the effect of endoscopic surgery. Quality assessment showed none of the trials were blind to patients, but five of the trials applied blinding in outcome assessment. Nearly half of the trials did not provide information on random sequence generation or allocation concealment (Supplement Figure 2).

**Figure 1:**
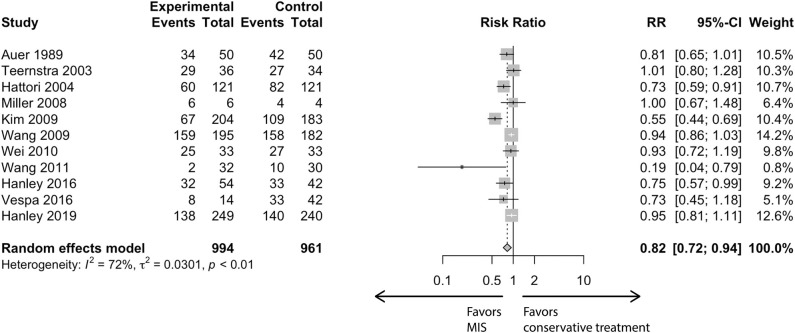
Conventional meta-analysis.

**Figure 2:**
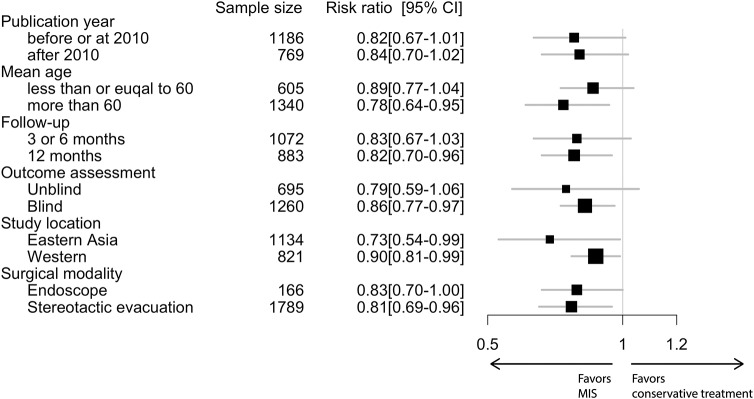
Subgroup analysis in conventional meta-analysis.

In the conventional meta-analysis, the risk ratios of MIS vs. conservative treatment were 0.82 [95% confidence interval (CI), 0.72–0.94] for the primary outcome (Figure 1) and 0.74 (95% CI, 0.62–0.88) for the secondary outcome. In subgroup analysis for the primary outcome, the point estimation kept relatively constant from 0.73 to 0.90 in regard to different subgroups (age, follow-up period, outcome blinding, publication year, study location or surgical modality; Figure 2).

**Figure 3:**
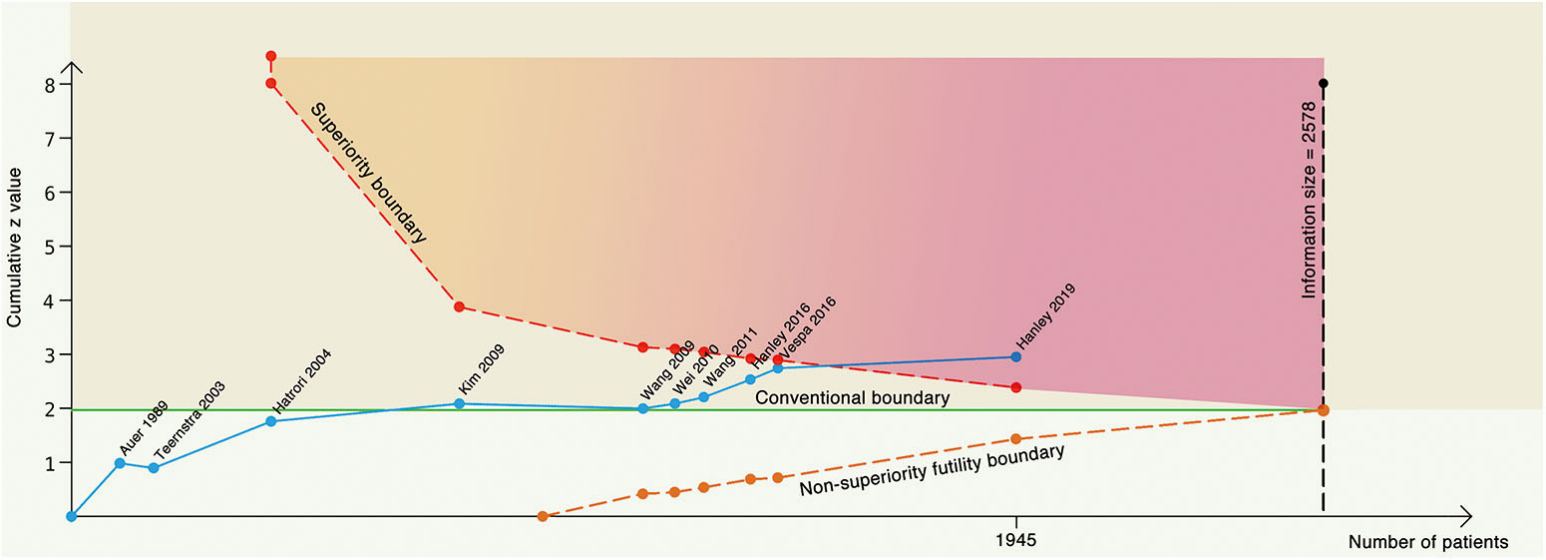
Trial sequential analysis with α = 5%, β = 80% to detect 18.8% relative risk reduction. The blue line represents the cumulative z line, the green line represents the conventional boundary, the red dotted line represents the superiority boundary, the orange dotted line represents the noninferiority futility boundary, and the black dotted line represents the acquired information size.

Figure 3 summarizes TSA results for the primary outcome. Analysis with the addition of the Vespa trial in 2016 (26) was inconclusive with a significant variation (95% CI, 0.61–1.01). After the addition of the latest Hanley trial (11), the cumulative *z* curve (*z* = 2.93) crossed the α spending superiority boundary (*z* = 2.37, dashed red line), although the required information size (2,578 patients) was not reached. The result confirmed an 18.8% relative risk reduction of MIS vs. conservative treatment with moderate confidence (α = 0.05, β = 80%). For the secondary outcome, it was also highly likely that MIS would reduce mortality by 24.3% with moderate confidence (α = 0.05, β = 80%, Table 1).

**Table 1:** Trial sequential analysis on primary and secondary outcome with different prior.

	**Relative risk reduction**	**Power**	**Incidence in the control**	**Heterogeneity (diversity)**	**Information size**	**Risk ratio**	**Boundary**	**Explanation**
**(A) TRIAL SEQUENTIAL ANALYSIS ON PRIMARY OUTCOME (PROPORTION OF PATIENTS WITH MODIFIED rANKIN SCORE** **>** **3)**								
Conventional meta-analysis with random-effects model						0.82 (0.72–0.94)		
TSA	18.8 (Estimated)	80%	67.4%	81.9% (Estimated)	2578, not reached	0.81 (0.69–0.96)	Superiority crossed	MIS better
	15	80%	67.4%	81.9% (Estimated)	3994, not reached	0.81 (0.66–1.01)	Superiority nearly cross	Inconclusive
	10	80%	67.4%	81.9% (Estimated)	8807, not reached	0.81 (0.65–1.02)	Superiority not crossed	Inconclusive
	18.8 (Estimated)	90%	67.4%	81.9% (Estimated)	3452, not reached	0.81 (0.67–0.99)	Superiority crossed	MIS better
	18.8 (Estimated)	80%	58.0% (Latest study)	81.9% (Estimated)	4885, not reached	0.81 (0.66–1.00)	Superiority crossed	MIS better
**(B) TRIAL SEQUENTIAL ANALYSIS ON SECONDARY OUTCOME (MORTALITY)**								
Conventional meta-analysis with fix-effects model						0.76 (0.64–0.89)		
TSA	24.3 (Estimated)	80%	25.3%	0.0% (Estimated)	1435, reached	0.76 (0.63–0.90)	Superiority crossed	MIS better
	20	80%	25.3%	0.0% (Estimated)	2157, not reached	0.76 (0.63–0.91)	Superiority crossed	MIS better
	15	80%	25.3%	0.0% (Estimated)	3898, not reached	0.76 (0.61–0.94)	Superiority crossed	MIS better
	10	80%	25.3%	0.0% (Estimated)	8956, not reached	0.76 (0.60–0.96)	Superiority crossed	MIS better
	24.3 (Estimated)	90%	25.3%	0.0% (Estimated)	1921, reached	0.76 (0.62–0.93)	Superiority crossed	MIS better
	24.3 (Estimated)	80%	25.3%	30.0%	2050, not reached	0.76 (0.64–0.90)	Superiority crossed	MIS better

*MIS, Minimal invasive surgery; TSA, Trial sequential analysis*.

In the sensitivity analysis with different prior (Table 1), the result would be inconclusive if the risk reduction were assumed to be 15 or 10%, because the cumulative *z* curve did not cross the monitoring boundary; neither had the required information size been reached. If we assume higher confidence (β = 90%) or a lower event proportion (58.0%) in the control group, both analyses would yield a crossed superiority boundary indicating the efficacy of MIS. For the secondary outcome, the evidence was sufficient to conclude MIS is better even if the effect size decreased to 10%, the power increased to 90%, or the heterogeneity increased to 30%.

**Figure 4:**
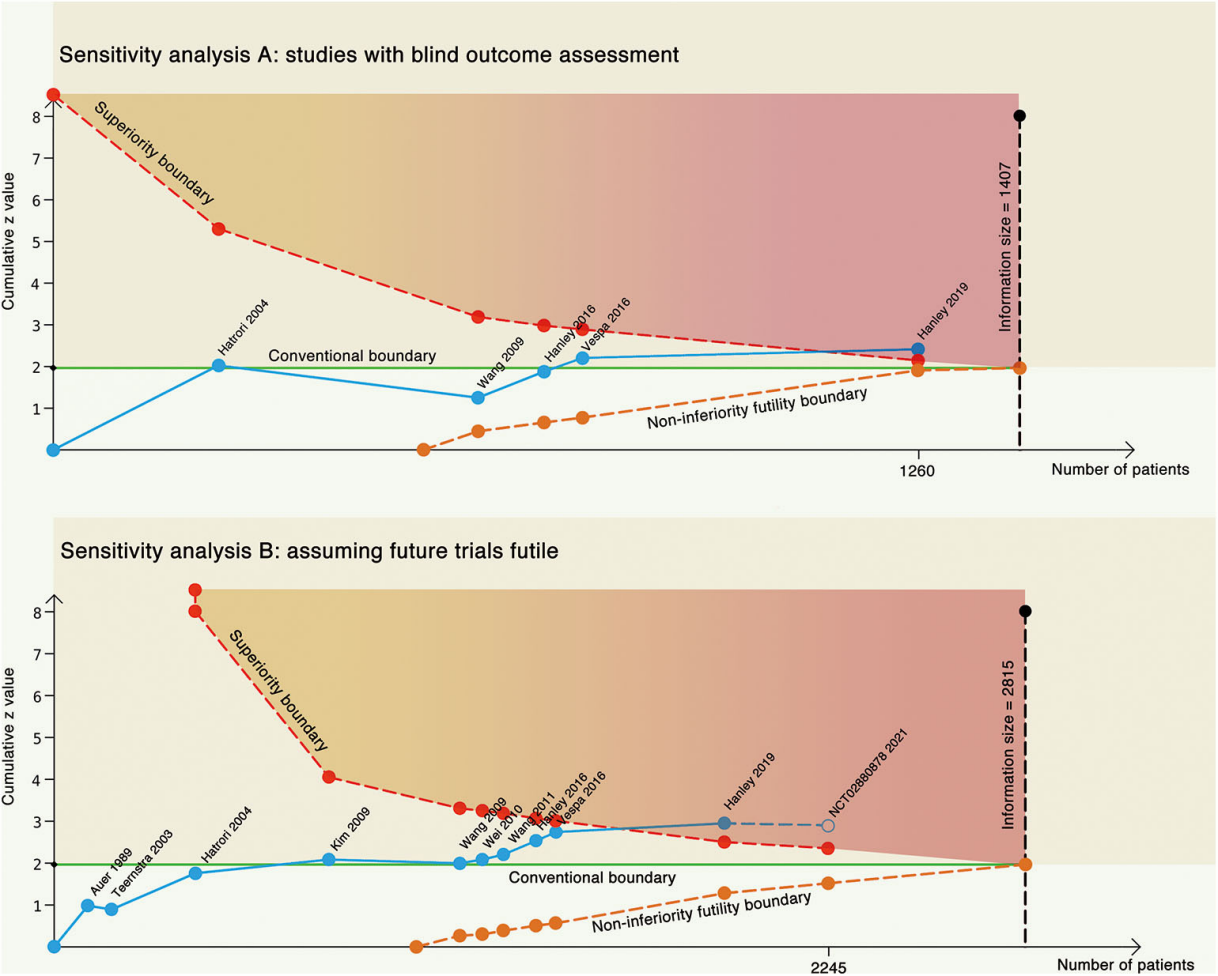
Two sensitivity analyses. **(A)** Trial sequential analysis on only studies with blind outcome assessment. **(B)** Trial sequential analysis on all the studies, assuming future trial futile. The blue line represents the cumulative z line, the green line represents the conventional boundary, the red dotted line represents the superiority boundary, the orange dotted line represents the non-inferiority futility boundary, and the black dotted line represents the acquired information size.

In another sensitivity analysis, we included only trials with blind outcome assessment (Figure 4A). The cumulative *z* score crossed the required information size with an adjusted risk ratio of 0.86 (95% CI, 0.73–1.02) using the same prior in the main analysis. We further assumed that the results of the ongoing RCT (NCT02880878: estimated 300 participants) are futile (in this case, both the treatment and control groups would have the same outcome incidence: 58%) to inspect the robustness of our results (Figure 4B). The cumulative *z* curve still stood above the superiority boundary that suggested our analysis might not be subjective in future trials.

## Discussion

Minimally invasive surgery demonstrated improved clinical outcome (the proportion of surviving patients without or with slight neurological debilitation) over conservative treatment in patients with intracerebral hemorrhage. The robust results with several sensitivity analyses indicated a sufficient amount of evidence favoring MIS such that further trials are unlikely to change the conclusion.

Several meta-analyses (5–10) have been published on the similar topic, but we argue these meta-analyses have their limitations compared to our approach, including a mixture control group, involving nonrandomized trials and comprising trials with selection bias (Table 2). The CI may shrink when combining many studies with different controls. Nonrandomized studies have the potential risk of selection bias, in which surgeons may select those patients with better outcome probability. Studies with both craniotomy and MIS in which the decision of craniotomy or MIS was made at the discretion of surgeons also introduced selection bias if only the MIS patients were included in the meta-analysis (4, 27). We also argue that our analysis answered the question of which treatment is better, especially in the circumstances that the latest trial (11) was futile, and the question whether we need more trials to compare MIS vs. conservative treatment in patients with intracerebral hemorrhage.

**Table 2:** Summary of previous published meta-amylases on the similar topic.

**References**	**Treatment**	**Control**	**Included studies**	**Primary outcomes**	**Limits**	**Conclusion**
Zhou et al. (9)	MIS	Conservative treatment or craniotomy	RCT	Death or dependence	The selection of MIS in Zuccarello Mendelow studies was biased; mixture control	MIS better
Akhigbe et al. (8)	MIS	Conservative treatment	RCT	Mortality	The selection of MIS in Zuccarello study was biased; only include five studies	Inconclusive
Yao et al. (7)	Endoscope	Stereotactic evacuation, conservative treatment or craniotomy	RCT + non-RCT	Mortality	Biased due to non-randomized studies; mixture control	Endoscope better
Xia et al. (6)	MIS	Craniotomy	RCT + non-RCT	Mortality	Biased due to non-randomized studies	MIS better
Tang et al. (5)	MIS	Conservative treatment or craniotomy	RCT + non-RCT	Death or dependence	Only include Eastern Asian patients; biased due to non-randomized studies; mixture control	MIS better
Scaggiante et al. (10)	MIS	Conservative treatment or craniotomy	RCT	Death or dependence	The selection of MIS in Zuccarello studies was biased; mixture control	MIS better

*MIS, Minimal invasive surgery; RCT, randomized control trial*.

Trial sequential analysis can avoid premature conclusion when meta-analyses based on traditional hypothesis testing would have falsely identified the effect as significant (12, 15). Another advantage of TSA is to estimate the sample size of future trials if the current result is inconclusive. The major limitation of applying TSA lies in that prespecified prior may have a significant impact on the result, which requires many sensitivity analyses to test the robustness.

We also calculated the required sample size of a single trial based on our meta-analysis: 554 patients are required to have an 80% chance of detection, to be significant at the 5% level, a decrease in the primary outcome measure from 67.4% in the control group to 55.9% in the experimental group. None of the current trials reached the required sample size including the latest published one. We also have to acknowledge the population diversity in calculating the sample size. Our subgroup analysis suggested the population might be an effective modifier with a higher effect in the Asian population. Although the mechanism was not clear, sample size calculation in future trials should incorporate the population information.

Because of the nature of comparing a surgical approach vs. a nonsurgical treatment, blinding of the patients was neither possible nor ethical, but several trials applied blinding to the outcome assessment. Our subgroup analysis shows that unblinded assessment had a larger effect size and a wider CI, suggesting the results were biased.

The effect of endoscopic surgery might be different from that of stereotactic evacuation. But several studies found that residue hemorrhage may be a risk factor for unfavorable outcomes in these patients (11, 28). The mean posttreatment day 1 hemorrhage volume reduction was roughly 30% in two stereotactic trials (11, 17). Only 59% of participants achieved the target hemorrhage volume reduction in the Hanley trial (11). In endoscopic trials, the compliance rate could be 95% (26), and the reduction of hemorrhage could be as high as 70% (26), which suggest a potential higher effect. For the per-protocol effect, the benefit of MIS would be larger in practice, considering the crossover rate is higher in the conservative group than in the surgical group (3, 27) as long as surgery was not inferior to conservative treatment.

Our study has several strengths. Our work added further evidence to the existing literature supporting MIS over conservative treatment in patients with intracerebral hemorrhage. Moreover, we answered the question whether we need more trials to save the cost of future unnecessary trials. Second, although the results of TSA were dependent on the prior, we used several sensitivity analyses to show that our results were robust at higher power assumption or within studies with high quality. We also included studies in both English and Chinese as there was geographic variation in the lifetime risk of stroke, with the highest risks in East Asia, Central Europe, and Eastern Europe (29, 30).

The major limitation of our study lies in the mixture of treatments as the differentiation between the two investigated treatments may lead to underpower. Comparative risks and effectiveness of different techniques have never been studied. Even trials using stereotactic evacuation were heterogeneous in applying different modalities. Another limitation of our study was the various definitions of outcome and follow-up time, which should be taken into account when interpreting these data. We were unable to answer the question which subgroup might benefit more from MIS, although we observed that mean age might be an effective modifier, which is consistent with the result in the latest trial (11).

## Conclusion

Minimally invasive surgery seems to be more effective than conservative treatment in patients with intracerebral hemorrhage in reducing both morbidity and mortality. Repeating a clinical trial with similar devices, design, and outcomes is unlikely to change current evidence. Future trials should target at comparative effectiveness among different approaches.

## Author Contributions

XZ and LX collected the data. YA did the analysis. XZ and NQ wrote the manuscript.

## Conflict of Interest

The authors declare that the research was conducted in the absence of any commercial or financial relationships that could be construed as a potential conflict of interest.
