# Supplementary material for: Assessment of Evidence Regarding Minimally Invasive Surgery vs. Conservative Treatment on Intracerebral Hemorrhage: A Trial Sequential Analysis of Randomized Controlled Trials
Source: Front Neurol. 2020 Jun 4;11:426. doi: 10.3389/fneur.2020.00426 (PMC7287205; doi:10.3389/fneur.2020.00426)

Supplement Table 1. PRISMA Checklist

**Section/topic**	**#**	**Checklist item**	**Page/Paragraph Location**
**TITLE**			
Title	1	Identify the report as a systematic review, meta-analysis, or both.	Page 1 Title
**INTRODUCTION**			
Rationale	3	Describe the rationale for the review in the context of what is already known.	Page 3 Introduction Paragraph 2-3
Objectives	4	Provide an explicit statement of questions being addressed with reference to participants, interventions, comparisons, outcomes, and study design (PICOS).	Page 3 Introduction Paragraph 3
**METHODS**			
Protocol and registration	5	Indicate if a review protocol exists, if and where it can be accessed (e.g., Web address), and, if available, provide registration information including registration number.	Page 3 Methods Paragraph 1
Eligibility criteria	6	Specify study characteristics (e.g., PICOS, length of follow-up) and report characteristics (e.g., years considered, language, publication status) used as criteria for eligibility, giving rationale.	Page 3 Methods Paragraph 1
Information sources	7	Describe all information sources (e.g., databases with dates of coverage, contact with study authors to identify additional studies) in the search and date last searched.	Page 3 Methods Paragraph 1
Search	8	Present full electronic search strategy for at least one database, including any limits used, such that it could be repeated.	Page 3 Methods Paragraph 1
Study selection	9	State the process for selecting studies (i.e., screening, eligibility, included in systematic review, and, if applicable, included in the meta-analysis).	Page 3 Methods Paragraph 1. (Supplement figure 1)
Data collection process	10	Describe method of data extraction from reports (e.g., piloted forms, independently, in duplicate) and any processes for obtaining and confirming data from investigators.	Page 4 Methods Paragraph 4, *data extraction section*
Data items	11	List and define all variables for which data were sought (e.g., PICOS, funding sources) and any assumptions and simplifications made.	Page 4 Methods Paragraph 2-4 *intervention, outcome and data extraction section*
Risk of bias in individual studies	12	Describe methods used for assessing risk of bias of individual studies (including specification of whether this was done at the study or outcome level), and how this information is to be used in any data synthesis.	Page 4 Methods Paragraph 4, *data extraction section*

Section/topic	#	Checklist item	Page/Paragraph Location
Summary measures	13	State the principal summary measures (e.g., risk ratio, difference in means).	Page 4 Methods Paragraph 5, *data synthesis section*
Synthesis of results	14	Describe the methods of handling data and combining results of studies, if done, including measures of consistency (e.g., I^2) for each meta-analysis.	Page 4 Methods Paragraph 5-6, *data synthesis section*
Risk of bias across studies	15	Specify any assessment of risk of bias that may affect the cumulative evidence (e.g., publication bias, selective reporting within studies).	Page 4 Methods Paragraph 7, *data extraction section*
Additional analyses	16	Describe methods of additional analyses (e.g., sensitivity or subgroup analyses, meta-regression), if done, indicating which were pre-specified.	Page 4 Methods Paragraph 7, *data synthesis section*
**RESULTS**			
Study selection	17	Give numbers of studies screened, assessed for eligibility, and included in the review, with reasons for exclusions at each stage, ideally with a flow diagram.	Page 5 Results Paragraph 1 (Supplement figure 1)
Study characteristics	18	For each study, present characteristics for which data were extracted (e.g., study size, PICOS, follow-up period) and provide the citations.	Page 5 Results Paragraph 1 (Supplement Table 2)
Risk of bias within studies	19	Present data on risk of bias of each study and, if available, any outcome level assessment (see item 12).	Page 5 Results Paragraph 1 (Supplement Figure 2)
Results of individual studies	20	For all outcomes considered (benefits or harms), present, for each study: (a) simple summary data for each intervention group (b) effect estimates and confidence intervals, ideally with a forest plot.	Page 5 Results Paragraph 2 (Figure 1)
Synthesis of results	21	Present the main results of the review. If meta-analyses are done, include for each, confidence intervals and measures of consistency	Page 5 Results Paragraph 2-3 (Table 1, Figure 2)
Risk of bias across studies	22	Present results of any assessment of risk of bias across studies (see Item 15).	Page 5 Results Paragraph 1 (Supplement figure 2)
Additional analysis	23	Give results of additional analyses, if done (e.g., sensitivity or subgroup analyses, meta-regression [see Item 16]).	Page 5 Results Paragraph 4-5 (Figure 3)
**DISCUSSION**			

Section/topic	#	Checklist item	Page/Paragraph Location
Summary of evidence	24	Summarize the main findings including the strength of evidence for each main outcome; consider their relevance to key groups (e.g., healthcare providers, users, and policy makers).	Page 6 Discussion Paragraph 1
Limitations	25	Discuss limitations at study and outcome level (e.g., risk of bias), and at review-level (e.g., incomplete retrieval of identified research, reporting bias).	Page 7 Discussion Paragraph 8
Conclusions	26	Provide a general interpretation of the results in the context of other evidence, and implications for future research.	Page 7 Conclusions Paragraph 1
**FUNDING**			
Funding	27	Describe sources of funding for the systematic review and other support (e.g., supply of data); role of funders for the systematic review.	N/A

Supplement Table 2. Detailed search strategy

Pubmed	(“intracerebral hemorrhage” or “intracranial hemorrhage” or “cerebral hemorrhage” or “brain hemorrhage” or “basal ganglia hemorrhage” or “thalamic hemorrhage” or “hemorrhagic stroke” and “hemorrhage” or “hematoma”) and (“minimally invasive” or “minimal surgical procedures” or “endoscopy” or “endoscopic” or “stereotaxic” or “stereotactic” or “aspiration” or “keyhole” or “craniopuncture” or "surgery")<br>Filters: **Randomized Controlled Trial**
Web of Science/CNKI	TS(“intracerebral hemorrhage” or “intracranial hemorrhage” or “cerebral hemorrhage” or “brain hemorrhage” or “basal ganglia hemorrhage” or “thalamic hemorrhage” or “hemorrhagic stroke” and “hemorrhage” or “hematoma”)<br>AND<br>TS=(“minimally invasive” or “minimal surgical procedures” or “endoscopy” or “endoscopic” or “stereotaxic” or “stereotactic” or “aspiration” or “keyhole” or “craniopuncture” or "surgery")

Supplement Table 3. Characteristics of included studies

Year	Author	Location	Treatment/control (Male: Female)	Age	Treatment	Outcome	Results
1989	Auer	Graz, Australia	50(28:22)/50(33:17)	46.1	Endoscope	6 months mRS	Futile
2003	Teernstra	Maastricht, Netherland	36(19:17)/34(21:13)	68.0	Stereotactic evacuation + urokinase	6 months mRS	Futile
2004	Hattori	Tokyo, Japan	121(71:50)/121(77:44)	60.5	Stereotactic evacuation	12 months ADL	Superior
2008	Luo	Guangzhou, China	36(21:15)/39(23:16)	55.3	Stereotactic evacuation + urokinase	6 months ADL	Superior
2008	Miller	Los Angeles, USA	6(6:0)/4(3:1)	59.0	Endoscope	3 months mRS	Futile
2009	Kim	Masan, Korea	204(257:47)/183(132:51)	65.8	Stereotactic evacuation	6 months mRS	Superior
2009	Wang	Beijing, China	195(114:81)/182(122:60)	56.7	Stereotactic evacuation + urokinase	14 days mRS	Superior
2010	Wei	Sichuan, China	36(19:17)/39(22:17)	57.0	Stereotactic evacuation	3 months GOS	Superior
2011	Wang	Tianjin, China	32(18:14)/30(16:14)	46.0	stereotactic evacuation + urokinase	6 months ADL	Superior
2016	Hanley	Multicenter	54(35:19)/42(28:14)	60.9	Stereotactic evacuation + urokinase	12 months mRS	Futile
2016	Vespa	Multicenter	14(9:5)/42(28:14)	61.0	Endoscope	12 months mRS	Futile
2019	Hanley	Multicenter	250(159:91)/249(146:103)	62.0	Stereotactic evacuation + alteplase	12 months mRS	Futile

mRS: modified Rankins Score; ADL: Activities of Daily Living; GOS: Glasgow outcome scale

Supplement Figure 1. PRISMA flow chart

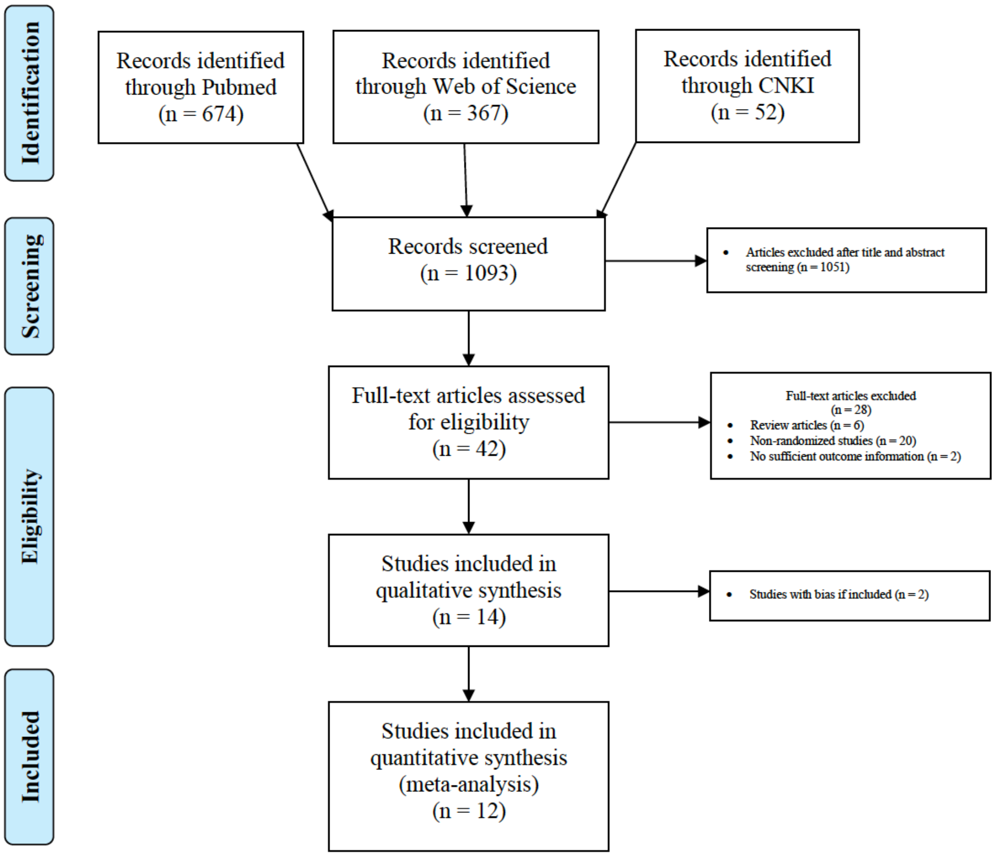

Supplement Figure 2: The risk of bias

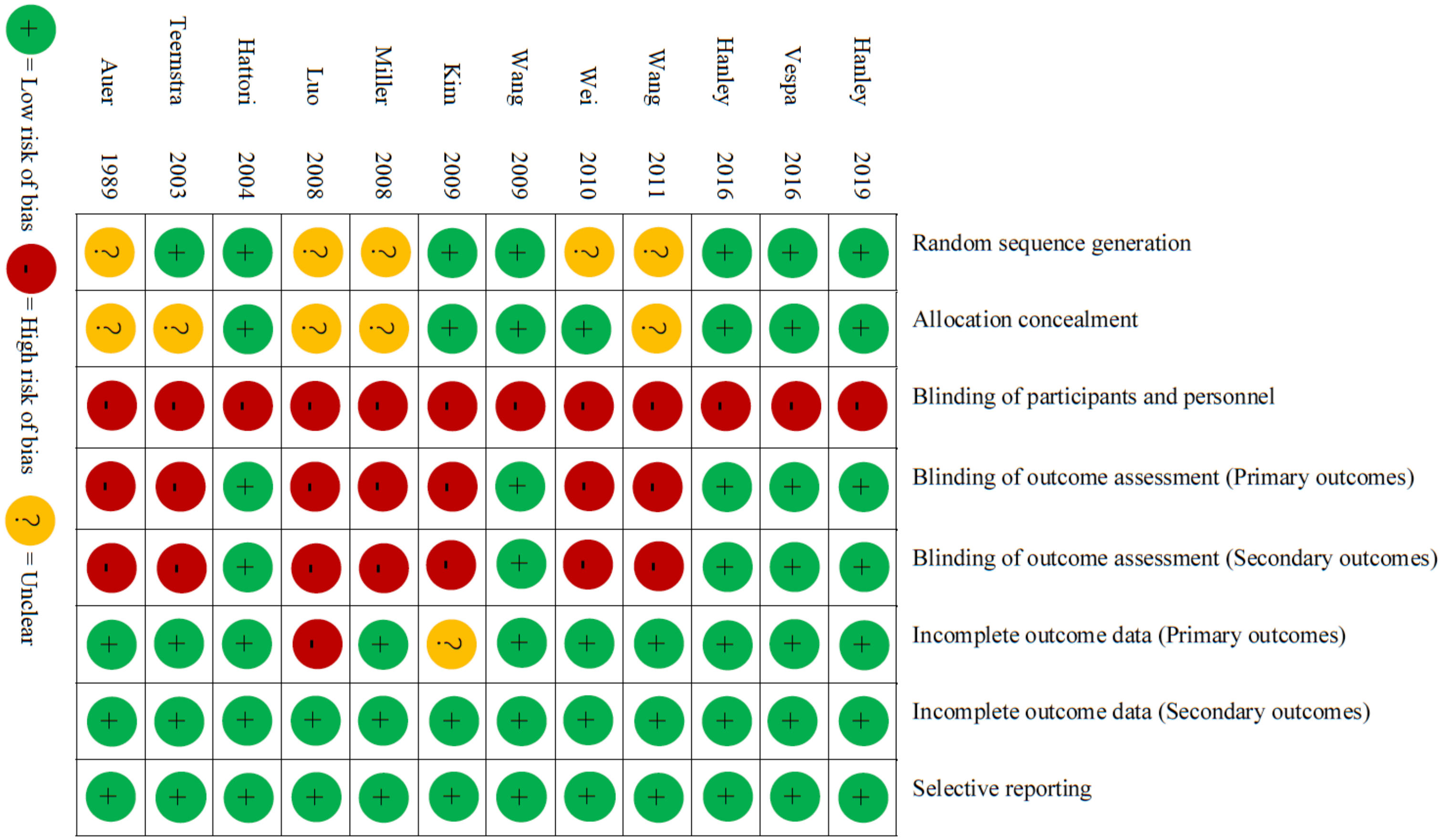

Supplement: Supplementary file 1 [file Data_Sheet_1.PDF]
